# Compensatory hypertrophy of the superior gluteus maximus following botulinum toxin-induced weakening of the inferior fibers

**DOI:** 10.1016/j.jpra.2025.12.016

**Published:** 2025-12-20

**Authors:** Kyu-Ho Yi, Jin-Hyun Kim, Isabella Rosellini, Jong Keun Song, Wong Ka Fai, Byung Ki Cho, Konstantin Frank, Ji-Soo Kim, Carlos Bautzer, Benjamin Ascher

**Affiliations:** a Division in Anatomy and Developmental Biology, Department of Oral Biology, Human Identification Research Institute, BK21 FOUR Project, Yonsei University College of Dentistry, Seoul, Korea; b You and I Clinic, Seoul, Korea; c Avery Beauty Clinic and Avena Aesthetics, Jakarta, Indonesia; d Pixelab Plastic Surgery Clinic, Seoul, Korea; e Gaddiel Medical Group Limited, Tsim Sha Tsui, Hong Kong, China; f It’s Me Clinic, Sejong, Korea; g Department of Plastic, Hand and Reconstructive Surgery, University Hospital Regensburg, Regensburg, Germany; h Private Practice, Ho Chi Mihn City, Vietnam; i Lifestyle Clinic, Sao Paulo, Brazil; j SIBUS-In, Paris, France

**Keywords:** Botulinum toxin, Gluteus maximus, Muscle hypertrophy, Chemodenervation, Body contouring

## Abstract

Selective chemodenervation of the inferior gluteus maximus (GMax) may induce compensatory hypertrophy of its superior fibers. Two healthy female subjects underwent ultrasound-guided botulinumtoxinA (JETEMA THE TOXIN, JETEMA Co., Ltd., Korea) injections (100 IU) into the inferior GMax. Six-week follow-up revealed increased fullness and definition in the superior region without adverse effects. The observation supports the concept that targeted muscle weakening can promote adaptive hypertrophy in adjacent fibers. This minimally invasive approach may have potential applications in aesthetic body contouring and functional rehabilitation.

## Introduction

Botulinum toxin type A (BoNT-A) is widely used for selective muscle weakening in both therapeutic and aesthetic contexts. While its use in facial muscles is well established, application to large postural muscles such as the gluteus maximus (GMax) remains relatively unexplored.^2^ We present two clinical observations demonstrating compensatory hypertrophy of the superior GMax following targeted BoNT-A injection into its inferior fibers.

## Clinical observation and procedure

The Gluteus Maximus (GMax) is composed of distinct fiber groups with specific functional roles; superior fibers primarily aid in hip abduction and external rotation, while inferior fibers drive hip extension.^1^ When one functional unit is weakened, adjacent fibers may undergo adaptive hypertrophy to compensate. We hypothesized that selective chemodenervation of the inferior GMax would increase the mechanical demand on the superior portion, triggering visible hypertrophy over time.

**Figure 1:**
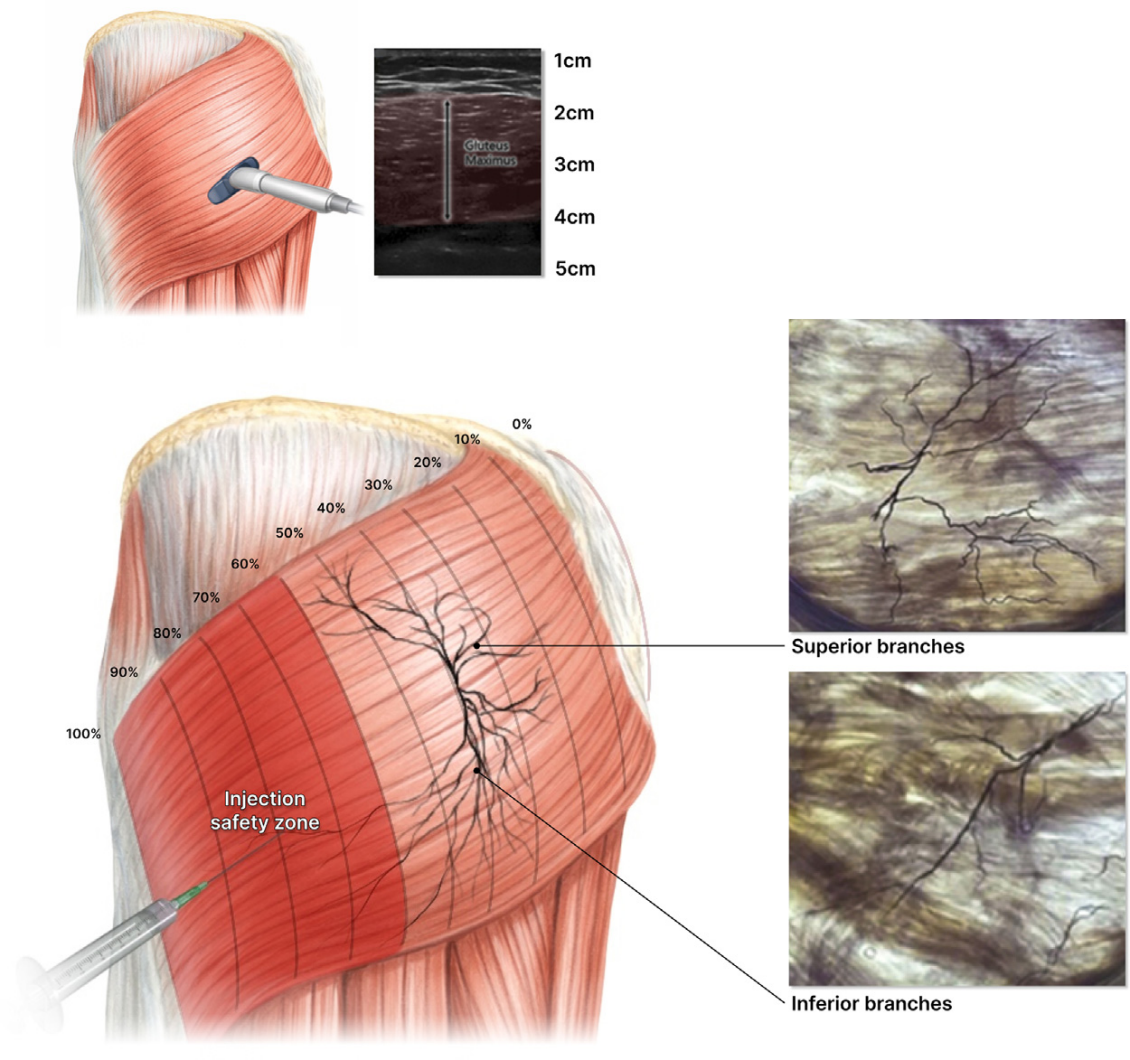
Composite schematic integrating anatomical dissection and ultrasound-guided injection mapping of the gluteus maximus. The illustration shows the intramuscular neurovascular arborization pattern of the superior and inferior branches with magnified insets highlighting regional vascular distribution. Ultrasound correlation demonstrates the injection pathway targeting the inferior fibers within the red-shaded 60–100 % zone of sparse innervation. Each red circle denotes a 10 U injection point (total 100 U).

Two healthy female subjects seeking upper gluteal enhancement underwent ultrasound-guided BoNT-A injection (botulinumtoxinA, (JETEMA THE TOXIN, JETEMA Co., Ltd., Korea)) into the inferior GMax. Injection mapping was based on intramuscular nerve arborization patterns (Figure 1).^3^ A total of 100 IU botulinumtoxinA was diluted in 5 mL saline (20 U/mL). Ten units were injected per point within the 60–100 % region along the muscle’s vertical axis, where innervation is sparse.^3^ The procedure was performed under topical anesthesia using a 1-inch needle to reach the deep inferior fibers. Post-procedure, patients avoided exercise for 48 h to minimize diffusion risk.^4^ No adverse events occurred.

**Figure 2:**
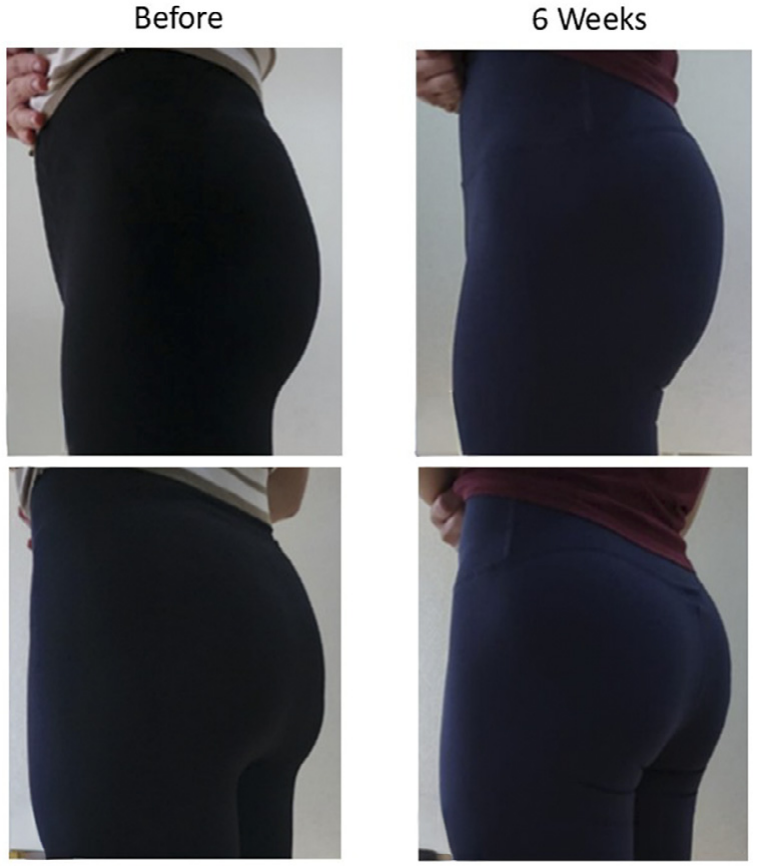
Representative clinical photographs of two female subjects before and 6 weeks after botulinum toxin injection. Post-treatment images demonstrate visible hypertrophy and enhanced contour of the superior gluteus maximus consistent with compensatory enlargement.

At 6 weeks, both subjects exhibited visible hypertrophy and improved definition of the superior GMax (Figure 2). Lateral and posterior photographs demonstrated fuller contours in the upper buttock, producing a subtle lifting effect. The volume increase remained stable for approximately 4 months before gradual attenuation. Functional strength and gait remained unchanged. No asymmetry or pain was reported.

## Discussion

BoNT-A acts by blocking acetylcholine release at the neuromuscular junction, causing temporary chemodenervation and reduced contractile efficiency of injected fibers. The resulting redistribution of load to neighboring motor units can induce compensatory hypertrophy. This mechanism has been described in facial and limb muscles but has not been reported in the gluteal region. Our findings suggest that this adaptive response is clinically detectable in the GMax as well.

From an aesthetic standpoint, this approach offers a minimally invasive option for selective modulation of gluteal contour without implants or fat grafting.^5^ In rehabilitation, a similar principle could help redirect activation to underused muscle segments in cases of imbalance or partial denervation.

Limitations include the small sample size, lack of objective volume measurements, and short follow-up. Further work using MRI quantification and electromyography could clarify the extent and duration of the effect. Controlled studies are needed to define optimal dose, injection pattern, and reproducibility.

## Conclusion

Targeted BoNT-A injection into the inferior gluteus maximus can induce compensatory hypertrophy of its superior fibers. This observation introduces a potentially novel method for selective muscle modulation in aesthetic and functional practice.

## Author contribution

Writing – Original Draft Preparation: Kyu-Ho Yi, Jin-Hyun Kim, Jong Keun Song. Writing – Review & Editing: Kyu-Ho Yi, Isabella Rosellini, Wong Ka Fai. Visualization: Kyu-Ho Yi, Byung Ki Cho, Konstantin Frank, Ji-Soo Kim. Supervision: Kyu-Ho Yi, Carlos Bautzer, Benjamin Ascher.

## Declaration of competing interest

None declared.
